# ATP-binding and hydrolysis of human NLRP3

**DOI:** 10.1038/s42003-022-04120-2

**Published:** 2022-11-03

**Authors:** Rebecca Brinkschulte, David M. Fußhöller, Florian Hoss, Juan F. Rodríguez-Alcázar, Mario A. Lauterbach, Carl-Christian Kolbe, Melanie Rauen, Semra Ince, Christian Herrmann, Eicke Latz, Matthias Geyer

**Affiliations:** 1 Institute of Structural Biology, University of Bonn, Venusberg-Campus 1, 53127 Bonn, Germany; 2 Institute of Innate Immunity, University of Bonn, Venusberg-Campus 1, 53127 Bonn, Germany; 3 Physical Chemistry I, Ruhr University Bochum, 44780 Bochum, Germany

**Keywords:** Biochemistry, Immunology

## Abstract

The innate immune system uses inflammasomal proteins to recognize danger signals and fight invading pathogens. NLRP3, a multidomain protein belonging to the family of STAND ATPases, is characterized by its central nucleotide-binding NACHT domain. The incorporation of ATP is thought to correlate with large conformational changes in NLRP3, leading to an active state of the sensory protein. Here we analyze the intrinsic ATP hydrolysis activity of recombinant NLRP3 by reverse phase HPLC. Wild-type NLRP3 appears in two different conformational states that exhibit an approximately fourteen-fold different hydrolysis activity in accordance with an inactive, autoinhibited state and an open, active state. The impact of canonical residues in the nucleotide binding site as the Walker A and B motifs and sensor 1 and 2 is analyzed by site directed mutagenesis. Cellular experiments show that reduced NLRP3 hydrolysis activity correlates with higher ASC specking after inflammation stimulation. Addition of the kinase NEK7 does not change the hydrolysis activity of NLRP3. Our data provide a comprehensive view on the function of conserved residues in the nucleotide-binding site of NLRP3 and the correlation of ATP hydrolysis with inflammasome activity.

## Abstract (web summary)

Analysis of the inflammasome-forming protein NLRP3 provides insights into the function of conserved residues in the ATP-binding site of NLRP3 and the correlation of ATP hydrolysis with inflammasome activation.

## Introduction

The innate immune system of higher organisms has evolved a strategy to recognize stressors and invading pathogens by its pattern recognition receptors (PRRs)^1,2^. To facilitate the detection of pathogens, it recognizes pathogen-associated microbial patterns (PAMPs), like lipopolysaccharide (LPS), or damage/danger-associated molecular patterns (DAMPs)^3^. DAMPs can be of proteinaceous origin, as for example heat-shock proteins, or comprise endogenous molecules like ATP, uric acid or deoxyribonucleic acid (DNA). Multi-protein complexes like inflammasomes recognize danger signals and invading pathogens by the core component, the NOD-like receptor (NLR). The best studied inflammasome-forming protein to date is NLRP3, a multidomain protein of 118 kDa in size that is composed of an N-terminal Pyrin domain (PYD), a central NACHT domain, and a C-terminal leucine-rich repeat (LRR) domain.

The formation of the NLRP3 inflammasome has been shown to depend on two consecutive signals: A priming event, initiating the transcriptional upregulation of canonical and non-canonical inflammasome components like NLRP3, and an additional activation event^4^. NLRP3 is activated by a multitude of diverse stimuli, converging in the common mechanism of ASC speck formation and subsequent activation of Caspase-1. The NLRP3 inflammasome assembly is based on the formation of a molecular platform, initiated by the oligomerization of NLRP3, which is thought to serve as a scaffold for the association of the adaptor protein ASC that is mediated by homotypic PYD–PYD interactions^5,6^. The filament formed by the ASC protein in turn serves as a binding platform for Caspase-1 that is recruited to the inflammasome via CARD–CARD interactions^5,7^.

NOD-like receptors belong to the family of ATPases associated with various cellular activities (AAA+ ATPases). Classification of this protein family starts with the discrimination of P-loop NTPases into the two major groups of Kinase-GTPases (KG) and the Additional Strand Catalytic E (ASCE) group^8^. These two main classes are distinguished by their substrate specificity, with members of the family belonging to the ASCE division, which includes the AAA+ ATPases, having a higher affinity for ATP^8,9^. Within the AAA+ ATPases, the NOD-like receptors are classified into the family of signal transduction ATPases with numerous domains (STANDs) including NB-ARC and NACHT NTPases, respectively^9–11^.

The function of AAA+ ATPases is diverse, ranging from proteases like the 26S proteasome over proteins involved in vesicular fusion to helicases and transcriptional activators^8,10,12^. AAA+ ATPases are found among all kingdoms of life. They are known to convert the energy generated during ATP-binding and hydrolysis into mechanical force that is used to evoke conformational changes of their substrates like proteins and nucleic acids^12,13^. A typical feature of AAA+ ATPases is the assembly into oligomers, like dimers, hexamers or open-ring structures^14^. For example, the NLR-related apoptosis inducing protein CED-4 assembles into a closed octameric ring structure^15^ whereas the active NLRC4 protein adopts a disc-like structure with up to 11 subunits or even spiral-like helical polymers^16–19^. Usually, ATP hydrolysis takes place in the assembled oligomer, like in the hexameric assembly of VpS4^20,21^.

In its nucleotide-binding domain, the superfamily of ASCE AAA+ ATPases contains a conserved alpha/beta Rossmann-like fold. Five parallel β-strands build the central element that are arranged in a distinct 5-1-4-3-2 order^11,22,23^. The β-strands harboring the main ATPase elements like the Walker A and B motifs on strands 1 and 3, respectively, are interconnected by loops and alpha helices. Among the subfamilies of ASCE, the core fold contains individual C-terminal extensions, like an additional helical bundle in AAA+ ATPases, the C domain or additional β-strands in RecA-like ATPases^10,12,14^. The C domain is the main feature identified to group STAND ATPases, including the NLRs, within the family of AAA+ related ATPases^10^.

Among the NLRP family members, interaction with ATP and ADP nucleotides has been experimentally determined for NLRP1^24–26^, NLRP3^27^, NLRP7^28^, NLRP10^29^, and NLRP12^30^, usually going along with an involvement in protein activation. Moreover, NLRP3 inflammasome formation was shown to be abrogated in the presence of the specific inhibitor CY-09, for which a direct interaction with the ATP-binding Walker A/B sites of NLRP3 was proposed^31^. Decreasing intracellular ATP levels have been correlated with NLRP3 inflammasome activation^32^. A recent computational analysis of NLRP1 to NLRP14 identified amino acid residues that may be involved in nucleotide binding^33^ and modeling of NLRP3 suggested enhanced ATP-binding and multimerization in disease related cryopyrin-associated periodic syndrome (CAPS) mutations^34^. Molecular dynamic simulations examined the active site of ADP- and ATP-bound NLRP3 models supporting distinctions in nucleotide-binding domain topology that disseminate on to the global protein structure^35,36^. However, a thorough experimental analysis of ATP hydrolysis activity of NLRP3 using analytical methods with highly purified protein has been missing to date. Likewise, experimental description of residues involved in nucleotide binding and their potential impact on ATP hydrolysis and cellular protein activation is pending. This study aims at the molecular characterization of ATP binding by NLRP3, yielding both biochemical and functional insights into the assembly of the NLRP3 inflammasome.

## Results

### Recombinant full length NLRP3 adopts two different conformational states

**Fig. 1:**
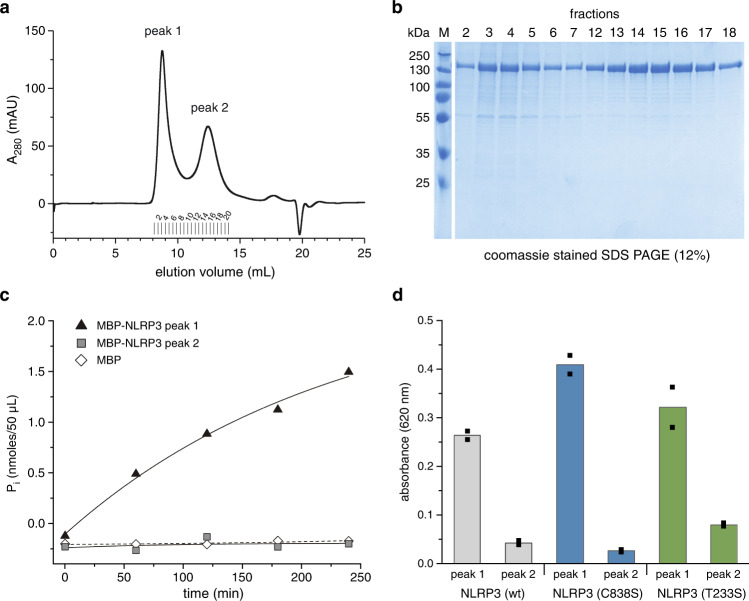
The two conformers of NLRP3 exhibit different ATP hydrolysis activity. **a** Human MBP-NLRP3, subjected to gel filtration analysis (Superose 6 10/300 GL), elutes in two distinct peaks, peak 1 and peak 2, respectively. **b** SDS-PAGE (12%) analysis of untreated MBP-NLRP3, according to gel filtration fractions shown in **a**. The protein band at about 165 kDa corresponds to the theoretical MW of MBP-NLRP3. **c** The malachite green phosphate assay was used to determine the ATP hydrolysis activity of MBP-NLRP3. The amount of P_i_ was measured in a time course experiment over 240 min. MBP-NLRP3 peak 1 and 2 (0.4 µM) were incubated at 20 °C in the presence of 1 mM ATP and 5 mM MgCl_2_. MBP was included as control; *n* = 1. **d** The ATP-hydrolysis activity of MBP-NLRP3 wt, C838S and T233S (0.4 µM), each showing peak 1 and peak 2, is compared. The amount of P_i_ generated in the presence of 1 mM ATP and MgCl_2_ was determined using the malachite green phosphate assay after 90 min incubation at 22 °C. Bars represent mean of technical duplicates shown as individual data points.

To characterize the ATP binding site of NLRP3, we expressed human MBP-tagged NLRP3 in baculo-virus infected *Sf9* insect cells and succeeded in generating soluble and pure wild type, full-length protein. When subjected to gel filtration chromatography, the protein eluted in two distinct peaks indicating the formation of two different oligomeric entities (Fig. 1a). One peak (peak 1) eluted close to the void volume indicative of a high molecular weight assembly. A second peak (peak 2) eluted at a size corresponding to a molecular weight larger than 670 kDa, suggesting the homomeric assembly of multiple subunits. The peaks could be separated by fractionation and remained stable upon purification. Both peaks contained only MBP-NLRP3 protein but no other contaminant or eluate from cellular expression as shown by Coomassie stained SDS PAGE analysis (Fig. 1b). Here, the protein migrates as expected from its molecular weight of 158 kDa. The protein was purified to homogeneity without any indication of degradation in both peak fractions (Supplementary Fig. 1). Post-translational modifications, particularly phosphorylations, were suggested to convey the inactive and active state of NLRP3^37,38^. Thus, the purified protein fractions were subjected to peptide mass fingerprinting spectrometry analysis. An enrichment of S198 phosphorylation in the active loop region was detected for peak 1 relative to peak 2, although the phosphorylation was not at stoichiometric levels.

In initial experiments, the ATP hydrolysis activity of NLRP3 was analyzed using the malachite green phosphate assay, which detects the release of inorganic phosphate P_i_. Using a standard protocol, protein samples of both peaks from MBP-NLRP3 were incubated in the presence of ATP and MgCl_2_ at 30 °C. At a concentration of 0.4 µM MBP-NLRP3 (peak 1), 1 mM ATP and 5 mM MgCl_2_, the malachite green phosphate assay revealed up to 1.50 nmoles/50 µL P_i_, corresponding to 29.9 µM, over a time period of 4 h (Fig. 1c). In contrast, no free P_i_ was detected for MBP-NLRP3 (peak 2) or the MBP control. Based on these data, an ATP turnover rate of 0.31 min^−1^ was calculated for MBP-NLRP3 peak 1, whereas no hydrolysis activity was seen for MBP-NLRP3 peak 2. The single-point mutations C838S and T233S showed a similar peak distribution in the elution profiles as the wild type NLRP3 protein when subjected to gel filtration chromatography analysis at 4 °C. Here again, the hydrolysis activity of the two mutants indicated by the release of P_i_ was much higher for peak 1 compared to peak 2 (Fig. 1d). These initial observations suggest differences in the nucleotide binding accessibility and hydrolysis activity of the two different structural states of NLRP3.

### NLRP3 hydrolyzes ATP with multi-cycle turnover kinetics

**Fig. 2:**
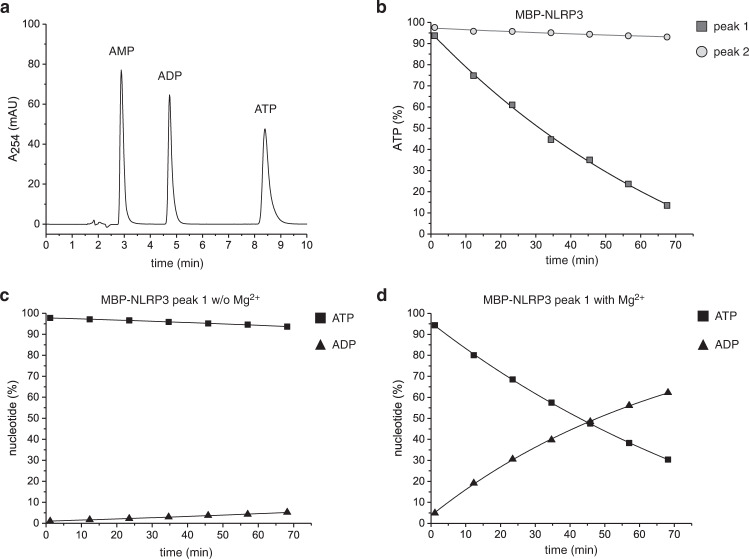
ATP-hydrolysis activity of NLRP3 determined by reverse-phase HPLC. **a** Separation of adenosine nucleotides by HPLC on a RP-18 column. Nucleotides elute at about 3 mL (AMP), 4.5 mL (ADP) and 8.5 mL (ATP). **b** MBP-NLRP3 peak 1 or peak 2 (3 µM) were incubated at 25 °C for 68 min in the presence of 5 mM MgCl_2_ and 100 µM ATP. The amount of AMP, ADP and ATP was determined in 10 min intervals using RP-HPLC. The eluted peaks were evaluated by integration and the total integral adjusted to 100%. The relative ATP concentration is shown on the y-axis and the time on the x-axis (min). Shown is one representative measurement of *n* > 5 biologically independent experiments. **c** The hydrolysis activity of MBP-NLRP3 peak 1 depends on MgCl_2_. MBP-NLRP3 peak 1 (3 µM) was incubated at 25 °C for 68 min in the presence of 100 µM ATP, but in the absence of MgCl_2_. Shown is one representative experiment of *n* = 2 independent experiments. **d** ATP is hydrolyzed by NLRP3 peak 1 to ADP. MBP-NLRP3 peak 1 at 3 µM concentration was incubated at 25 °C for 68 min in the presence of 5 mM MgCl_2_ and 100 µM ATP. The relative amount of nucleotide is shown for ATP and ADP. Shown is one representative measurement of *n* > 5 biologically independent experiments.

The ATP-hydrolysis activity of recombinant NLRP3 was analyzed by reverse phase (RP) HPLC to determine ATP turnover rates and to characterize the ATP binding site. In RP-HPLC chromatography, the nucleotides are separated according to their charge, enabling the precise calculation of tri- and di-phosphate ratios at given time points of the hydrolysis reaction by nucleotide peak integration (Fig. 2a). We set up a multi-cycle turnover kinetics experiment using 3 µM NLRP3 protein, separated by gel filtration into peak 1 or peak 2, and 100 µM ATP co-substrate, following the reaction course over 68 min at 25 °C. Time points were taken every 10 min followed by the automatic injection lasting 65 s. First, we used the HPLC setup to monitor ATP hydrolysis activity and to determine hydrolysis rates for MBP-NLRP3 using 5 mM MgCl_2_ in the reaction buffer. As seen before in the malachite green phosphate assay, MBP-NLRP3 peak 1 showed hydrolysis activity for ATP, whereas peak 2 showed only residual activity, exhibiting approximately a 14-fold reduced turn-over number compared to peak 1 (Fig. 2b). As a control, the dependence on MgCl_2_ for the hydrolysis of ATP by NLRP3 was analyzed. Now, MBP-NLRP3 peak 1 showed only a very moderate hydrolysis activity for ATP in the absence of MgCl_2_, indicating the dependence on a Mg^2+^ ion for the hydrolysis reaction to occur (Fig. 2c). Furthermore, the onset of ADP was seen at an amount as ATP declined in RP-HPLC experiments (Fig. 2d). Under the conditions chosen, the ATP hydrolysis reaction appeared largely linear over time, indicating the higher affinity of ATP over ADP for NLRP3. The calculated turn-over numbers of ATP hydrolysis were determined from the initial, linear part of the reaction only and are listed in Table 1.

**Table 1:** ATP turnover numbers of human NLRP3 proteins.

NLRP3 protein	turnover (min^−1^)	fold change relative to wt peak 1
Native		
wt (peak 1)	0.385	1.00
wt (peak 2)	0.027	0.07
Walker A		
K232A	0.132	0.34
T233S	0.464	1.21
Walker B		
D302A	0.074	0.19
G303E	0.046	0.12
D305A	0.071	0.18
E306Q	0.086	0.22
D305A/E306A	0.084	0.22
D305A/E306Q	0.161	0.42
G303E/D305A/E306A	0.136	0.35
Sensor 1		
R351T	0.037	0.10
R351G	0.154	0.40
Sensor 2		
H522D	0.065	0.17
H522R	0.331	0.86
M523A	0.589	1.53
P-motif		
P412A	0.077	0.20
Glu-switch		
R262W	0.204	0.53

The ATP-hydrolysis reaction of full length MBP-NLRP3 and protein mutants (always peak 1) was analyzed over time by RP-HPLC using 3 µM NLRP3 protein, 100 µM ATP and 5 mM Mg^2+^ at 25 °C. For wild type NLRP3, peak 2 was also measured under the same conditions. The resulting turnover numbers (min^−1^) derived from the initial linear part of the curves are listed.

Using RP-HPLC we also tested whether recombinantly expressed and purified MBP-NLRP3 protein harbors a bound nucleotide when purified in absence of nucleotide in the buffer solution. Neither of the two NLRP3 protein samples fractionated from peak 1 or peak 2, respectively, showed even at high protein concentrations of 100 µM in the RP-HPLC measurement a peak profile corresponding to bound nucleotide (Supplementary Fig. 2). Thus, MBP-NLRP3 peak 1 and 2 are considered to have an empty nucleotide binding pocket upon affinity purification and gel filtration chromatography. This suggests a low binding affinity of NLRP3 for ATP or ADP as similarly observed, e.g., for kinases but differently from GTP-binding proteins like the small GTPase Ras^39^. Finally, purine GTP or pyrimidines CTP and UTP were tested as co-substrates for the hydrolysis activity of NLRP3 peak 1. Yet, these nucleotides did not show any degradation over time, suggesting that they are not incorporated into the ATP-binding site of NLRP3 (Supplementary Fig. 3).

### Mutational analysis of Walker A/B nucleotide-binding motifs in NLRP3

**Fig. 3:**
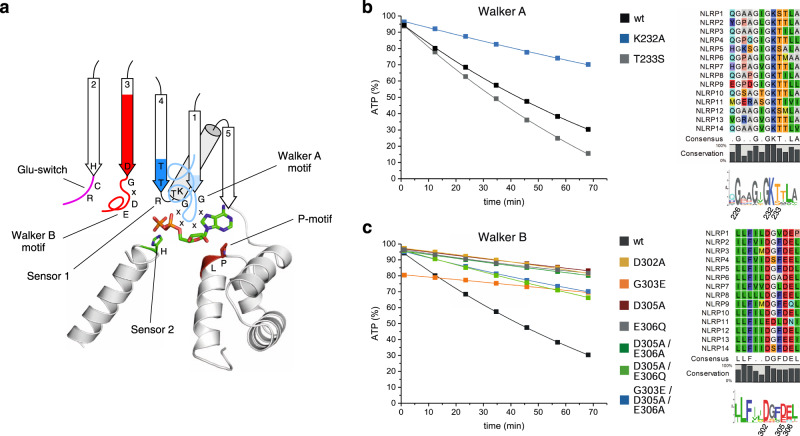
Mutational analysis of Walker A/B nucleotide-binding motifs in NLRP3. **a** A cartoon of the nucleotide-binding site of NLRP3 including ADP is shown based on the structure of inactive NLRP3 (PDB: 7PZC). The Walker A and B motifs as well as the Glu-switch, Sensor 1, Sensor 2 and the P-motif are indicated. **b** MBP-NLRP3 wild type and Walker A motif point-mutants were analyzed by RP-HPLC. Sequence alignments and sequence logos of the labeled motifs and residues are shown for human NLRP1-14. Protein samples (3 µM) were incubated at 25 °C in the presence of 100 µM ATP and 5 mM MgCl_2_. The amount of ATP (%) is shown on the y-axis and the time (min) on the x-axis. The amount of ATP was determined in 10 min intervals using RP-HPLC, integrated and normalized to 100% nucleotide. **c** MBP-NLRP3 wild type and Walker B motif point-mutants were analyzed by RP-HPLC using the same set-up as in **b**. All mutant significantly reduced the hydrolysis activity.

As part of the AAA+ ATPase superfamily, NLRP proteins contain a series of conserved sequence motifs that mediate nucleotide binding. Based on the crystal structures of the NACHT domain from NLRC4^40^ and NOD2^41^, and the cryo-EM structure of NLRP3 bound to NEK7^42^, we assigned residues in close proximity to ATP, showing the canonical motifs in their position relative to the bound diphosphate (Fig. 3a). The Walker A motif of all 14 NLRP proteins unifies to GxxGxGKT from the regular consensus sequence GxxxxGK(T/S), as shown in the sequence logo (Fig. 3b). The lysine residue typically forms electrostatic interactions with both the β- and γ-phosphate of the bound ATP molecule, whereas the hydroxyl group of the threonine side chain coordinates the magnesium ion. The K232A mutant reduced the ATP hydrolysis activity of NLRP3 by 3-fold, possibly due to a reduced ATP-binding affinity. Surprisingly, the T233S mutant instead showed a by 20% increased turnover number compared to the wildtype protein (Fig. 3b).

In NLRPs, a significant deviation in sequence conservation is found for the Walker B motif, located at the tip of β-strand 3 in the ATPase fold. The typical hhhhDE consensus motif, where h denotes a hydrophobic residue, is degenerated to hhhhDGxDE in NLRs. The glutamic acid in the typical Walker B motif is supposed to coordinate a water molecule that can be activated for the in-line attack of the γ-phosphate to initialize hydrolysis. In AAA+ ATPases, the glutamic acid at the tip of β-strand 3, is often slewed in by the ‘Glu-switch’-residues of the neighboring β-strand 2, transferring a signal from the terminal β-strand to the bound nucleotide^43^. The DE motif in NLRPs instead is located one helical turn downstream of the tip of the β-strand but still positioned in face to the terminal nucleotide phosphate group (Fig. 3a).

We generated several single point mutations in the Walker B motif of NLRP3 as well as double and triple combinations in order to reconstitute a typical Walker B motif in NLRP3. All mutant proteins were purified to homogeneity and analyzed by gel filtration chromatography (Supplementary Fig. 1). Off note, most proteins besides wild type NLRP3 and the T233S mutant eluted on a Superose 6 column in one single peak running close to the void volume. The single point mutations D302A, D305A, E306Q, and the double mutation D305A/E306A showed an at least 4.5-fold reduced hydrolysis activity compared to the wild type NLRP3 protein. The G303E mutation, resembling a bona fide Walker B motif, is the least hydrolysis active with an 8.4-fold reduced turnover number (Fig. 3c). The D305A/E306Q and G303E/D305A/E306A double and triple mutants instead exhibited an only 2.4- and 2.8-fold reduced hydrolysis activity, respectively, suggesting a cooperative effect of the DGxDE motif in maintaining the hydrolysis activity of NLRP3 (Table 1). Overall, residues D302, G303, D305, and E306 could all be found as critical residues for ATP hydrolysis in NLRP3, giving rise to an altered but functional Walker B motif in NLRPs.

### Mutational analysis of sensory and conserved ATP-binding motifs in NLRP3

**Fig. 4:**
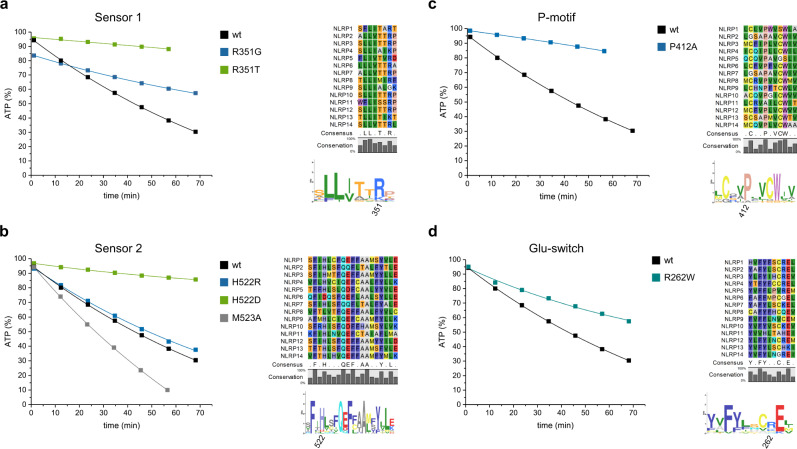
Mutational analysis of sensory ATP-binding motifs in NLRP3. **a** MBP-NLRP3 wild type and two different Sensor 1 mutants were analyzed by RP-HPLC. The protein samples (3 µM) were incubated at 25 °C in the presence of 100 µM ATP and 5 mM MgCl_2_. The relative amount of ATP (%) is shown on the y-axis and the time (min) on the x-axis. The amount of ATP was determined in 10 min intervals, integrated and normalized to 100% nucleotide. Sequence alignments and sequence logos of the labeled motifs and residues are shown for human NLRP1-14. **b** Hydrolysis analysis of MBP-NLRP3 Sensor 2 mutants performed similarly as in **a**. **c** ATP hydrolysis analysis of the P412A mutant. **d** ATP hydrolysis analysis of the R262W CAPS/Glu-switch mutant.

Two motifs in NLRPs are thought to sense the nucleotide-binding status of the inflammasomal protein. In AAA+ ATPases, the Sensor 1 residue located at the tip of β-strand 4, centered between the Walker A and B motifs (Fig. 3a), is usually a polar residue such as threonine^11^. This residue is altered to arginine (R) within a conserved TTR motif in members of the NLRP family (Fig. 4a). The positively charged arginine could form electrostatic interactions with the terminal phosphate group of the bound nucleotide and might shift a succeeding helix lying behind the central β-strands upon incorporation of a larger ATP molecule, thus leading to a conformational rearrangement in the NBD to HD2 subdomain assembly. In addition, Sensor 1 is described to assist the glutamate residue of the Walker B motif in the coordination of the attacking water molecule for ATP hydrolysis^44^. We tested two mutations of the sensory arginine for their effect on ATP hydrolysis. The threonine residue (R351T) was introduced as present in NLRC4, and a glycine (R351G), thus providing excessive space within the nucleotide binding site. The threonine mutation showed indeed the lowest hydrolysis rate of all mutations tested with a more than 10-fold decreased activity compared to the wild type protein (Fig. 4a). In contrast, the ATPase activity of the variant R351G was only 2.5-fold reduced. The introduction of a small glycine residue compare to the arginine possibly increases the ability to accommodate additional water molecules in the active site. Overall, the Sensor 1 residue R351 in NLRP3 can be confirmed as being crucial for ATP hydrolysis. Of note, the significantly reduced hydrolysis activity of the R351T mutant is also an indication that no major contaminants from the purification process are distorting the measurements.

The Sensor 2 motif has been identified in the crystal structure of NLRC4 and the cryo-EM structure of the NLRP3-NEK7 complex as a highly conserved histidine residue at the beginning of a central helix in the WHD subdomain, directly facing with its imidazole side chain the α- and β-phosphate groups of the bound ADP nucleotide^40,42^. As the electro-negativity of the phosphate-ester bonds determines the stability of the nucleotide towards hydrolysis, we analyzed the influence of both, a negatively and a positively charged residue at this position, and introduced a H522D and H522R mutant in NLRP3. RP-HPLC measurements revealed an ATP turnover number similar to the wild type protein for the positive charge mutant H522R, whereas introduction of a negative charge as naturally found at this position in NLRP6 diminished the ATP hydrolysis activity about 6-fold (Fig. 4b). A methionine residue, adjacent to the histidine, is exclusively found in NLRP3. In a computational analysis, M523 was suggested to take part in the ATP-binding of NLRP3^33^. M523 was therefore point-mutated to alanine and analyzed for its effect on ATP hydrolysis by RP-HPLC. The point-mutant M523A showed an increase in ATP hydrolysis activity by about 50%, compared to the wild-type protein (Fig. 4b). Thus, both H522 and M523 could be identified as important residues for ATP hydrolysis activity in NLRP3, expected to substitute for the function of a typical Sensor 2 motif in AAA+ ATPases.

An additional conserved proline residue was identified by sequence analysis in the HD1 domain of NLRPs, facing the adenine base of the bound nucleotide. Substitution of P412 by alanine led to a 5-fold decreased ATP hydrolysis turnover number compared to the wild-type protein (Fig. 4c). Although the proline residue is not directly participating in the coordination of the phosphate groups, it may significantly contribute to nucleotide binding, e.g., by sensing for the adenine base.

Besides its function in the NLRP3 associated disease CAPS, the point-mutation R262W was correlated to the predicted position of a Glu-switch residue found among AAA+ ATPases (Fig. 3a). Glu-switch residues, typically asparagine, serine or threonine, in the terminal β-strand 2 were found to control for the orientation of the glutamate residue located in the adjacent Walker B motif on β-strand 3, thus regulating the switch between inactive and active conformations^43^. In order to investigate the effect of the point-mutation R262W on the ATP hydrolysis of NLRP3, the CAPS mutation was tested in RP-HPLC experiments. Strikingly, the mutant does not lead to increased ATP hydrolysis activity, but has a by half reduced ATP turnover number compared to the wild-type protein. (Fig. 4d). Hence, both CAPS associated mutations R262W as well as D305A, which is located within the Walker B motif of NLRP3 (Fig. 3c), show reduced ATP hydrolysis activity. Therefore, the hyperactive NLRP3 inflammasome activity in CAPS disease might be due to a prolonged ATP residence time.

### Cellular characterization of canonical AAA+ ATP-binding motifs in NLRP3

**Fig. 5:**
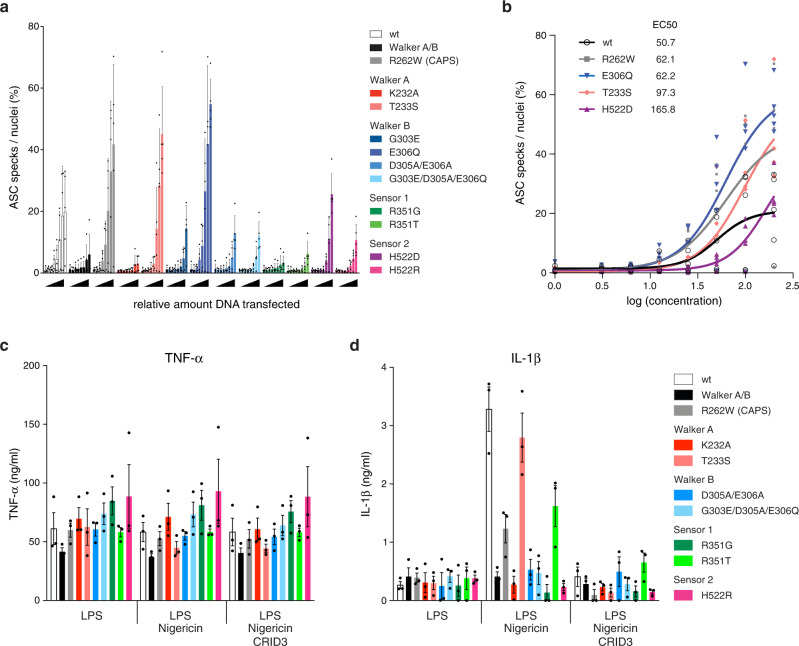
Cellular activity assays for NLRP3 mutants targeting the AAA+ ATPase fold. **a** Increasing amounts of different NLRP3 variants targeting the AAA+ ATPase fold were transiently over-expressed in HeLa cells stably over-expressing ASC-mTurquoise. ASC specks and nuclei were quantified using microscopy, and the ratio ASC speck/nuclei was plotted as a measure of inflammasome activation. Bars represent mean and SD of 5 independent experiments. **b** Dose response curves for the active NLRP3 mutants described in **a**. The half maximal effective concentration (EC50) was calculated for each of the NLRP3 variants. Data points of 5 independent experiments are shown. **c**, **d** NLRP3-deficient immortalized macrophages were reconstituted with wildtype NLRP3-mCitrine or indicated variants. **c** TNF-α and **d** IL-1β secretion of LPS primed immortalized macrophages stimulated with nigericin in the presence or absence of CRID3. Means ±SEM of pooled data from 3 independent experiments are presented.

NLRP3 requires the adaptor protein ASC to interact with Caspase-1 and assemble an inflammasome. Inflammasome formation can be visualized at the cellular level by monitoring ASC distribution in the cell^45,46^. ASC stays evenly distributed throughout the cytoplasm of cells during the steady state. Upon inflammasome activation, ASC is recruited to a single multiprotein complex in the cell and can be visualized by microscopy as a round structure of 0.5–1 µm termed ASC speck. ASC speck formation has therefore been used as a tool to study inflammasome assembly. To assess, whether NLRP3 mutants targeting the AAA+ ATPase fold are able to assemble an inflammasome, we transiently over-expressed increasing amounts of the NLRP3 mutants in HeLa cells that stably over-expressed ASC-mTurquoise. We then measured the capacity of each NLRP3 mutant to induce ASC specks. As observed by others, over-expression of wild type NLRP3 is sufficient to cause ASC speck formation in our system (Fig. 5a, b). Overexpression of similar amounts of the NLRP3 CAPS mutant R262W resulted in an exacerbated formation of ASC specks, whereas over-expression of a Walker A/B dead NLRP3 version (G231A/ K232A/ T233A/ D302A/ D305A/ E306A) was inefficient at triggering an ASC speck response (Fig. 5a, b). Overexpression of the mutant T233S, which conserves a canonical Walker A motif, elicited an ASC speck response similar to WT NLRP3, whereas overexpression of the Walker A motif dead mutant K232A failed to elicit the formation of ASC specks at any of the assayed concentrations.

Overexpression of the mutant E306Q, targeting the Walker B motif, showed increased ability to induce ASC specks in comparison to wild type NLRP3 (Fig. 5a, b). However, other mutations covering the Walker B motif (G303E, D305A/E306A, and even G303/D305A/E306Q) rendered NLRP3 unable to assemble an inflammasome (Fig. 5a). Overexpression of NLRP3 mutants targeting either Sensor 1 (R351T or R351G) or Sensor 2 (H522D or H522R) either failed at eliciting inflammasome assembly at any of the assayed concentrations (Fig. 5a) or at least showed an overall diminished response.

In macrophages, NLRP3 inflammasome activation requires two consecutive steps. During the first step, also called priming, NF-κB activation promotes transcription and translation of genes involved in inflammasome activation, i.e., NLRP3 and IL-1β. After activation, NLRP3 binds to pro-caspase-1 through ASC, promoting auto-activation of pro-caspase-1 and subsequent processing of pro-IL-1β and Gasdermin D. NLRP3 inflammasome activation culminates in secretion of IL-1β and commitment to a pro-inflammatory way of cell death termed pyroptosis.

To further investigate the functionality of NLRP3 mutants targeting the AAA+ ATPase fold in a more physiologically relevant cell type, we stably over-expressed NLRP3-mCitrine tagged variants in Nlrp3^-/-^ immortalized macrophages. We could successfully over-express the Walker A mutant K232A, the Walker B mutant G303/D305A/E306Q and the Sensor 1 mutants R351G and R351T at similar levels to wild type NLRP3 (Supplementary Fig. 4). To assess their capacity to secrete IL-1β upon activation, we primed the macrophage cell lines using LPS and stimulated them with NLRP3 or AIM2 inflammasome activators. Both, TNF-α secretion upon LPS treatment and IL-1β release in response to the AIM2 inflammasome activator poly(dA:dT) were similar in all cell lines analyzed (Fig. 5c and Supplementary Fig. 4b, c). However, with the exception of the Sensor 1 mutant R351T, all mutants were unable to secrete IL-1β upon treatment with the canonical NLRP3 activators nigericin or Leu-Leu-OMe (Fig. 5d and Supplementary Fig. 4c).

We were able to over-express the Walker A mutant T233S, the Walker B mutant D305A/E306A, the Sensor 2 mutant H522R and the CAPS mutant R262W only at lower levels than wild type NLRP3 (Supplementary Fig. 4c). Even though the expression level of the Walker A mutant T233S was lower than the one for wild type NLRP3, treatment of both cell lines with the NLRP3 activators nigericin or Leu-Leu-OMe promoted similar release of IL-1β (Fig. 5d and Supplementary Fig. 4e). Of note, when normalizing the IL-1β secretion against the expression level of the respective NLRP3 variant, T233S showed a much higher activity than wild type NLRP3 (Supplementary Fig. 5a, b). This observation backs the data obtained from the specking assays and points toward the idea that the Walker A mutation T233S results in a hyperactive NLRP3 variant. Pre-treatment of cells with the NLRP3 inhibitor CRID3 abolished IL-1β release elicited by any of the NLRP3 variants (Fig. 5d and Supplementary Fig. 4c).

A comparison of relative ATP hydrolysis, IL-1β secretion and ASC specking numbers for NLRP3 mutant proteins normalized to wild type protein is listed in Table 2 and Supplementary Fig. 5c. Of note, we were unable to stably over-express the Walker B mutant E306Q or the Sensor 2 mutant H522D in Nlrp3^−/−^ immortalized macrophages. According to our specking results with these mutants, this might reflect a scenario in which these two NLRP3 variants are hyperactive and already over-expression at early times promotes inflammasome activation and cell death via pyroptosis.

**Table 2:** Correlation of ATP hydrolysis with downstream signaling.

NLRP3 protein	ATP hydrolysis	IL-1β secretion	ASC specking
wt	1.00	1.00	1.00
R262W	0.53	0.38	1.78
K232A	0.34	0.08	0.15
T233S	1.21	0.85	1.54
D305A/E306A	0.22	0.16	0.27
R351G	0.40	0.04	0.15
R351T	0.10	0.49	0.13
H522R	0.86	0.07	0.26

Mean values of IL-1β secretion levels of LPS-primed immortalized macrophages and ASC specking (speck/nuclei) determined in HeLa cells transfected with 100 ng of DNA and reconstituted with indicated NLRP3 variants in comparison to ATP turnover numbers of recombinantly expressed NLRP3 variants, all normalized to wild type levels.

### NEK7, CRID3 and CY-09 do not inhibit NLRP3 hydrolysis activity

**Fig. 6:**
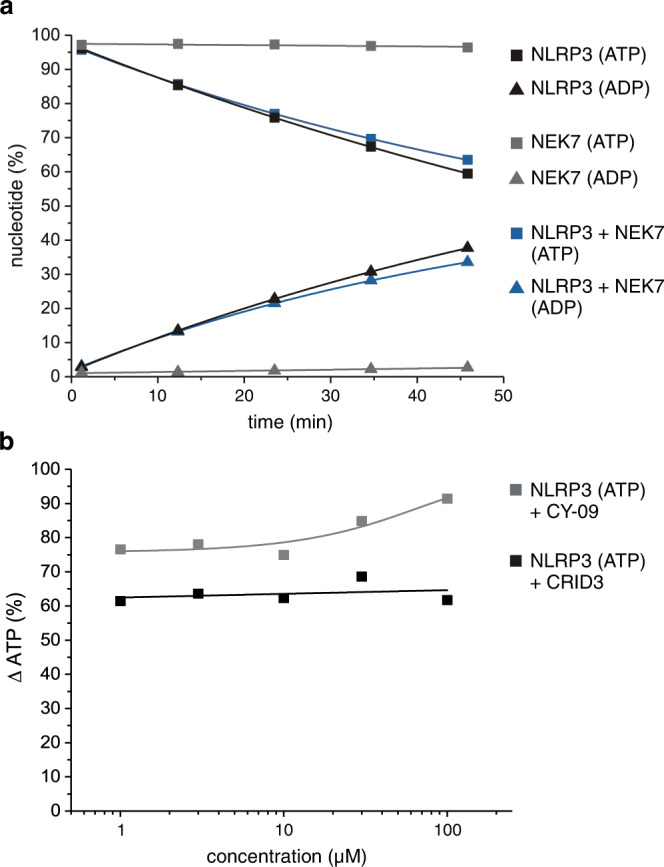
Effects of NEK7 and antagonists CRID3 (MCC950) or CY-09 on the ATP-hydrolysis activity of NLRP3. **a** The hydrolysis activity of MBP-NLRP3 peak 1 is unaffected by NEK7. MBP-NLRP3 peak 1 (3 µM) was incubated with NEK7 at 25 °C for 60 min in the presence of 100 µM ATP. The amount of ADP and ATP (%) is shown for MBP-NLRP3 peak 1 alone and addition of NEK7 to MBP-NLRP3. As a control, the kinase NEK7 exhibits no residual ATPase activity in the absence of substrate (gray symbols). **b** The hydrolysis activity of MBP-NLRP3 peak 1 is unchanged by CRID3 (MCC950) or CY-09. MBP-NLRP3 peak 1 (3 µM) was incubated at 25 °C for 60 min in the presence of 100 µM ATP. Increasing concentrations of CRID3 or CY-09 were added in a dose-response measurement from 1 to 100 µM and the relative concentration of ATP after 60 min measured.

The NIMA-related serine/threonine kinase NEK7 has been shown to directly interact with murine and human NLRP3 via the LRR-domain^42,47,48^. Complex formation of NEK7 with NLRP3 is thought to depend on K^+^ efflux, representing a consequence for NLRP3 activation^48,49^. The NLRP3 inflammasome assembly, including the formation of ASC specks, was found to be dependent on the association of NEK7 to the protein complex, but independent on its kinase activity^48^. To test the influence of NEK7 on ATP hydrolysis of NLRP3, MBP-NLRP3 peak 1 was incubated with NEK7 in a 1:1 molar ratio in the presence of 100 µM ATP and 5 mM MgCl_2_. As before, a time course experiment monitoring nucleotide turnover was recorded by RP-HPLC. The presence of NEK7 does not change the hydrolysis activity of NLRP3 as nearly identical amounts of ADP are produced in the presence or absence of the kinase (Fig. 6a). In a control experiment, the kinase NEK7 was incubated in the presence of 100 µM ATP and 5 mM MgCl_2_, in order to test for its intrinsic ATP turnover activity. As expected, and similarly seen for other kinases when substrate is missing, NEK7 does not exhibit residual ATP hydrolysis activity by itself (Fig. 6a).

Small-molecule inhibitors targeting the NLRP3 inflammasome promise a therapeutic benefit for a wide range of immune diseases including the CAPS mutant associated Muckle-Wells-Syndrome^50^. CRID3 (MCC950) is a small molecule inhibitor, which selectively inhibits non-canonical and canonical NLRP3 activation in the nanomolar concentration range^51^. Therefore, the effect of CRID3 on the ATPase activity of NLRP3 was tested by RP-HPLC measurements to clarify the underlying mechanism of action. Recombinantly expressed and purified MBP-NLRP3 peak 1 at a concentration of 3 µM was incubated with 100 µM ATP and 5 mM MgCl_2_ in the presence of increasing concentrations of CRID3 following a dose response titration series from 1 to 100 µM. In order to monitor the relative nucleotide hydrolysis over time, the samples were subjected to RP-HPLC measurements after 60 min incubation time (Fig. 6b). The amount of ATP hydrolyzed after 60 min reaction time remained constant for all five CRID3 concentrations tested, which was also equivalent to the DMSO control. Thus, addition of CRID3 to MBP-NLRP3 peak 1 does not influence the ATPase activity of the protein.

The small molecule CY-09 was recently described as a novel inhibitor of NLRP3 function^31^. The inhibitor was shown to directly interact with NLRP3 at a dissociation constant of ~0.5 µM. To study the effect of CY-09 on the hydrolysis activity of NLRP3, RP-HPLC measurements were performed. MBP-NLRP3 peak 1 was incubated in the presence of 100 µM ATP, 5 mM MgCl_2_ and increasing concentrations of CY-09 (Fig. 6b). Concentrations of up to 10 µM CY-09 did not result in a change in ATP-hydrolysis activity of NLRP3. Higher concentrations of up to 100 µM CY-09 instead even lead to a slight increase in ATP hydrolysis activity, suggesting, e.g., a potential stabilization of the protein. This stimulating effect of the NLRP3 ATPase activity however, is restricted to very high concentrations of CY-09. Nonetheless, a decrease in ATPase activity of MBP-NLRP3 was not confirmed for neither of the tested antagonistic compounds.

## Discussion

**Fig. 7:**
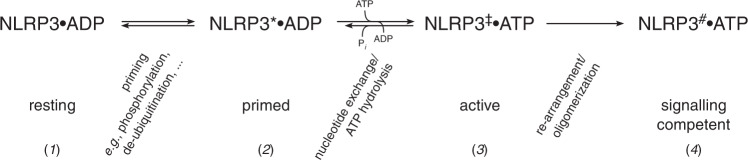
Schematic representation of the proposed activation mechanism of human NLRP3. From an inactive, resting state (1) the protein is turned, for example, by reversible PTMs such as phosphorylation, into a primed state (2) characterized by loss of autoinhibition. This state is capable of ATP uptake, which converts the protein to an active state (3). The ability of intrinsic ATP hydrolysis allows the protein to relapse to its resting or primed state. The active protein is eventually able to assemble into a quaternary structure, state (4), competent for downstream signaling, such as the induction of ASC filament formation.

Here we show that recombinant, full-length NLRP3 appears in two different entities of different sizes that can be separated by gel filtration analysis. While the smaller oligomeric complex (peak 2) is largely inactive, the high molecular weight entity (peak 1) has intrinsic ATP hydrolysis activity exceeding the turnover of a single nucleotide. The two different entities may correspond to an auto-inhibited, inactive state (peak 2) that is unable to uptake ATP in its nucleotide binding site, and a primed or active state (peak 1) readily interacting with nucleotides. A recent structural analysis revealed a homomeric decamer assembly for peak 2 of human NLRP3 that is bound to ADP and could be stabilized by the antagonist CRID3 whereas the conformations of peak 1 were found to be hetergeneous^52^. The intrinsic ATP-hydrolysis activity of NLRP3 measured in the multi-turnover assay is not only determined by the accessibility of the nucleotide binding site but also by the affinity of the protein for the nucleotide and the integrity of the catalytic center. A schematic representation of the proposed states in the activation mechanism of human NLRP3 is shown in Fig. 7. From an inactive, resting state (e.g., peak 2) the protein is turned, for example, by reversible PTMs such as phosphorylation, into a primed state (e.g., peak 1) characterized by loss of autoinhibition. This state is capable of ATP uptake, which converts the protein to an active conformation. However, the ability of ATP hydrolysis allows the protein to relapse to the resting or primed state. The active protein is eventually able to assemble into a quaternary structure competent for downstream signaling, such as the induction of ASC filament formation. This last step in the activation cascade of NLRP3 might be achieved by the assembly of the PYDs into a nucleation seed for ASC PYD filament elongation^53^.

As expected, mutation of the catalytic lysine in the Walker A motif reduces the ATP hydrolysis activity significantly. The loss in ATP hydrolysis activity is accompanied by a deficiency in ASC-speck formation, and IL-1β levels have been shown to be unaltered by LPS and nigericin treatment in cells. The Walker B motif is degenerated to hhhhDGxDE in NLRs with the C-terminal DE motif being important for the hydrolysis reaction as shown here. The role of the glutamate residue in the extended DGxDE motif of the NOD subfamily of STAND ATPases might be to activate a water molecule by making the oxygen more electronegative and hence a better nucleophile for attack of the γ-phosphorus. In contrast, restoring a bona fide Walker B motif (hhhhDE) largely decreases the ATP hydrolysis activity (Table 1). Again, a decrease in ATP hydrolysis activity, determined for the tested mutants, is shown to go along with the loss in ASC-speck formation and unaltered IL-1β levels in response to LPS and nigericin stimulation in cells. Interestingly, the mutant E306Q instead showed the strongest possible cell death effect ever observed in our laboratories. This might be indicative for the importance of the hydrolysis reaction of NLRP3, as the E306Q mutant protein might never acquire the resting, autoinhibitory state after ribosomal translation of mRNA into protein.

The two sensory motifs of NLRP3 according to AAA+ ATPase conformity, R351 and H522, are indeed important for protein activity and phosphate group recognition. The Sensor 1 mutation R351T not only severely diminishes ATP hydrolysis, but also fully abolishes the ability to form ASC specks in cells. Whereas we know today more than 210 single point mutations in human NLRP3 from clinical assessments that show an inflammatory active or hyperactive phenotype (see, e.g., the infevers database collection^54^) we hardly identify any non-responsive variants. The R351T mutation might therefore represent such rare loss-of-function phenotype for NLRP3 inflammasome formation. Furthermore, the R351G mutant showed the lowest potential for NLRP3 activation with respect to IL-1β release using different kinds of stimulation by either LPS only for the priming step or LPS plus nigericin or Leu-Leu-OMe (Fig. 5). This confirms the important function of Sensor 1 in the transmission of the activation signal, possibly by a conformational rearrangement of the NLRP3 subdomains irrespective of the ATP hydrolysis activity.

At the position of Sensor 2 (H522), replacement of the histidine by a positively charged arginine did not alter the ATP hydrolysis activity of NLRP3, whereas mutation to a negatively charged aspartic acid significantly decreased ATP turnover. Finally, GTP or pyrimidines are not tolerated as co-substrates in the ATP binding site of NLRP3, indicating the specificity for nucleotide recognition of this large ATPase^27^. Incubation of the ATP-binding site mutants with CRID3 (MCC950) in cells completely inhibited the IL-1β release in response to LPS and nigericin treatment, but strikingly the ATP hydrolysis activity of NLRP3 peak 1 was unaffected upon addition of CRID3 to recombinant protein. Hence, CRID3 is assumed to inhibit the activation of NLRP3 by a mechanism different than interfering with the ATP hydrolysis activity. Indeed, CRID3 was reported to close the active conformation of NLRP3 to an inactive state^55^ and to interact with NLRP3 at a site proximal to the Walker B motif^56^. These experimental observations were confirmed by recent structural analyses, showing CRID3 binding into a cleft in between the NACHT and the LRR domains in proximity to the nucleotide binding site^52,57^. CRID3 might therefore not be able to interact with NLRP3 peak 1, which has a readily accessible ATP binding site. Similarly, we could not confirm CY-09 as an ATP-competitive inhibitor of NLRP3 activity and the hydrolysis activity remained unchanged.

We here analyzed in a multi-turnover assay the hydrolysis activity of NLRP3. But how is the entry of ATP into the active site of NLRP3 regulated? On a molecular level, the activation mechanism of NLRP3 is not yet understood. It remains a conundrum, that NLRP3 requires ATP for its activation, which is the most abundant co-factor in cells, but does not simply get activated by high levels of cellular ATP. This resistance requires tight regulation either by co-factors or by an auto-inhibitory mechanism. The cryo-EM structures of mouse and human NLRP3 bound to ADP showed that NLRs might reside in an autoinhibited state with the FISNA domain closing the nucleotide binding site^52,58^. This suggests a closed state in the subdomain assembly that prevents ATP uptake. The two serine residues of the S_198_PVS_201_PIK sequence motif in the activation loop of human NLRP3 are indeed in close proximity to the residues of the FISNA domain that close the ATP-binding site. These serines were shown to be phosphorylated upon priming^38^, and consistent with such regulatory function, we observed an enrichment of S198 phosphorylation in peak 1 of the full-length protein expressed in insect cells^52^. Such phosphorylation mark could trigger a conformational change in the protein that ultimately leads to the appearance of two separated entities with different accessibilities for the bound nucleotide. Interestingly, NLRP1 was recently shown to interact with double-stranded RNA, leading to the activation of the intrinsic ATP hydrolysis of this inflammasomal protein^26^. It remains open, if RNA binding of NLRP1 goes along with a conformational change that might lead to increased ATP binding and hence the observation of higher ATP turnover rates.

The intrinsic ATP hydrolysis activity of wild type, full length NLRP3 must be considered rather low compared to other ATP- or GTP-binding proteins. The nucleotide exchange in AAA+ ATPases is supposed to correlate with large conformational movements of the N- and C-terminal subdomains relative to each other that are propagated through the oligomeric assembly of multiple subunits, often combined with the exertion of mechanical force^11,14^. One particular example is the human 26S proteasome, where different substrate engagement modes in the multimeric assemblies generate a cycle of asymmetric ATP hydrolysis around the ATPase motor^59,60^. For STAND ATPases however, we are only at the beginning of understanding how nucleotide binding and hydrolysis activity correlate with the conformational states and their transitions. The residence time of NLRP3 in the triphosphate-bound state might correlate with the time needed to execute these molecular movements. It is, however, of interest that point mutations in NLRP3 that diminish the ATP hydrolysis activity, as e.g., the CAPS mutation R262W or the E306Q mutant, lead to hyperactive inflammasome formation. Interfering with the nucleotide-binding site of NLRP3 by locking the protein in a diphosphate-bound state might thus be a strategy for potential anti-inflammatory intervention.

## Methods

### Protein expression and purification

The coding sequence of wild type or mutant human NLRP3 (aa 3-1036) was PCR-amplified and inserted into the pACEBac1 acceptor vector, modified to harbor an N-terminal MBP-tev affinity purification tag. Transformation was carried out into *E.coli* DH10 MultiBacTurbo cells and the recombinant bacmid DNA was used for transfection of *Sf9* insect cells to generate the initial virus stock V0. Passaging and subculturing of *Sf9* insect cells was carried out as suspension culture in SF-900TM III SFM medium (Thermo Fisher Scientific Inc., Waltham, USA) at 27 °C and 80 rpm, lacking antibiotics. The cell density and viability were continuously monitored using an automated cell counter EVE (VWR International, Vienna, Austria) and maintained at 0.3–3.0 × 106 cells/mL. The recombinant bacmid DNA was transfected into *Sf9* insect cells using the transfection reagent Cellfectin (Thermo Fisher Scientific Inc., Waltham, USA) according to the manufacturer’s instructions. The initial virus stock was amplified for 2 passages. The *Sf9* insect cells were grown in suspension culture to a cell density of 1.5 × 10^6^ cells/mL, supplemented with 4% Virus V2 and incubated for further 72 h at 27 °C at 80 rpm.

Cells were lysed in pre-chilled lysis buffer (20 mM Tris pH 8.0, 150 mM NaCl, 5 mM ßME) supplemented with DNase and subsequently sonicated. The cleared lysate was subjected to MBP affinity chromatography using a MBPtrap column (GE Healthcare, Munich, Germany), pre-equilibrated with lysis buffer. The protein was eluted in lysis buffer supplemented with 10 mM maltose. The protein was subsequently subjected to gel filtration chromatography using a Superose 6 10/300 GL increase column (GE Healthcare, Munich, Germany) in the following buffer: 20 mM HEPES pH 8.0, 150 mM NaCl and 1 mM TCEP.

The coding sequence of NEK7 (aa 26–302) was PCR-amplified and inserted into a pACEBac1 vector, modified to harbor an N-terminal GST-tag followed by a TEV protease cleavage site. Transformation into *E.coli* DH10 MultiBacTurbo cells and transfection of *Sf9* insect cells was carried out as described above. The cells were lysed in pre-chilled lysis buffer (50 mM HEPES pH 7.5, 300 mM NaCl, 5 mM ßME, 1 mM EDTA, 5% glycerol) supplemented with DNase and subsequently sonicated. The cleared lysate was subjected to GST affinity chromatography using a GSTrap column (GE Healthcare, Munich, Germany), pre-equilibrated with lysis buffer. The protein was eluted in lysis buffer supplemented with 10 mM GSH. The protein sample was incubated with TEV-protease in a ratio of 1:100 at 4 °C overnight. Subsequently, the protein was subjected to gel filtration chromatography using a HiLoad 16/600 Superdex 200 pg column (GE Healthcare, Munich, Germany) in the following buffer 50 mM HEPES pH 7.5, 150 mM NaCl, 1 mM EDTA, 1 mM TCEP and 5% glycerol.

### Malachite green phosphate assay

The malachite green phosphate assay kit (Cayman Chemical, Ann Arbor, USA) was used to measure the amount of inorganic phosphate (P_i_) generated during an ATP hydrolysis reaction, according to the manufacturer’s instructions. The proteins to be tested were diluted to a final concentration of 0.4 µM using a 10x buffer containing 200 mM HEPES pH 8.0, 1.5 M NaCl, 50 mM MgCl_2_, and incubated with ATP (Jena Bioscience, Jena, Germany) for 30 min (25 °C) or over a time course of 240 min (20 °C). Absorbance at 620 nm was measured using a Tecan plate reader (Tecan Trading AG, Männedorf, Switzerland). The standard curve and the protein samples were measured in technical duplicates.

### Ion-paired reverse phase HPLC

Ion-paired Reverse Phase HPLC was used to analyze the amount of nucleotide released upon an ATP hydrolysis reaction. Nucleotides and nucleotide analogs were separated using a Chromolith Performance RP-18 endcapped 100–4.6 HPLC column and the corresponding guard cartridge Chromolith RP-18 (Merck, Darmstadt, Germany). The measurement was performed using an Agilent 1260 infinity PSS bio-inert HPLC System (Agilent Technologies, Inc., Santa Clara, USA). The mobile phase was composed of the following: 10 mM tetrabutylammonium bromide, 30 mM K_2_HPO_4_, 70 mM KH_2_PO_4_, 0.2 mM sodiumazide, 4% acetonitrile, pH 6.5. The proteins to be tested were diluted to a final concentration of 3 µM in a buffer containing 20 mM HEPES pH 8.0, 150 mM NaCl, 5 mM MgCl_2_ and were incubated with 100 µM ATP (JenaBioscience, Jena, Germany) at 25 °C. The samples were incubated in glass vials (Waters Corporation, Milford, USA). 10 µl aliquots were taken every 10 min. The HPLC measurement was performed at 25 °C at a flow rate of 1 mL/min and the absorbance was continuously detected at 280 nm and 254 nm, respectively. The peak borders were set manually and the peak areas were integrated using the internal PSS software and normalized to 100%, enabling the calculation of nucleotide ratios. The hydrolysis data presented refers to individual protein preparations (*n* = 1). Regression curves represent exponential growth fittings.

Small-molecule inhibitors CRID3 (MCC950) and CY-09 were dissolved in DMSO. MBP-NLRP3 peak 1 (3 µM) was incubated at 25 °C for 60 min in the presence of 100 µM ATP, 5 mM MgCl_2_ and increasing concentrations, ranging from 0 µM to 100 µM, of CRID3 and CY-09, respectively. The DMSO concentration was kept constant for each of the samples.

### ASC specking assays

5 × 10^3^ HeLa cells stably over-expressing ASC-mTurquoise were seeded and transfected in duplicates with increasing amounts (0, 3.1, 6.3, 12.5, 25, 50, 100 or 200 ng) of plasmids encoding different variants of NLRP3-mCitrine fusion protein. 36 h after transfection, cells were fixed and nuclei were stained using a PBS solution containing both paraformaldehyde (4%) and DRAQ5 (1:2000). 10 images per well were taken using filter sets to detect mTurquoise (i.e., filter set 47 from Zeiss: excitation BP 436/20, beamsplitter FT 455, emission BP 480/40) and DRAQ5 (i.e., filter set 50 from Zeiss: excitation BP 640/30, beamsplitter FT 660, emission BP 690/50) using the 20x objective of a Zeiss observer Z1 microscope. Images were analyzed using CellProfiler 2.2.0 software to count nuclei and ASC specks. For each well, the ratio ASC specks/nuclei was calculated. Ratios extracted from 20 independent images (10 images per well, conditions in duplicates) were averaged to calculate the final ratio ASC speck/nuclei for every condition.

### Generation of NLRP3-mCitrine expressing iMOs

Immortalized NLRP3 deficient macrophages (iMOs) were generated by transducing NLRP3 deficient bone marrow derived macrophages with a J2 recombinant retrovirus, carrying the v-myc and v-raf(mil) oncogenes as described^61^. NLRP3-deficient iMOs were retrovirally transduced with constructs for the indicated NLRP3-mCitrine mutants. After retroviral transduction, cells were flow cytometrically sorted for mCitrine expression^37^. NLRP3-mCitrine expressing iMOs were cultured in DMEM containing 10% FCS and Penicillin-Streptomycin.

### NLRP3 activation of retrovirally transduced iMOs

NLRP3-mCitrine expressing iMOs were primed with 200 ng/ml LPS for 3 h. Supernatants were taken for priming control. 10 min before NLRP3 activation, cells were treated either with 10 μM CRID3 or serum free medium. Afterwards cells were challenged with following concentrations of NLRP3 activators: nigericin 10 μM, Leu-Leu-OMe 1 mM, or the AIM2 activator poly(dA:dT) 2 μg/ml. Poly(dA:dT) was transfected using 2.5 μl Lipofectamine 2000 per μg of poly(dA:dT). Supernatants were harvested after 1.5 h (nigericin) and 3 h (Leu-Leu-OMe and poly(dA:dT)), respectively. Secretion of TNFα and IL-1β was determined using HTRF kits by Cisbio Bioassays, Codolet, France.

### Statistics and reproducibility

For the malachite green phosphate assay, the results are presented as individual measurement or as bars representing the mean of technical duplicates shown as individual data points. For ATP-hydrolysis measurements of wt NLRP3 by reverse-phase HPLC, one representative measurement is shown of *n* > 5 biologically independent experiments. For mutant NLRP3 proteins representative individual measurements are shown. Cellular activity assays for NLRP3 mutants targeting the AAA+ ATPase fold are presented as means ± SEM of pooled data from at least 3 independent experiments. The effect of NEK7 and the antagonists CRID3 (MCC950) and CY-09 on the ATP hydrolysis activity of NLRP3 was determined in individual experiments.

### Reporting summary

Further information on research design is available in the Nature Research Reporting Summary linked to this article.

## Supplementary information

Supplementary Information

Description of Additional Supplementary Files

Supplementary Data 1

Reporting Summary

## Data Availability

The authors declare that all data supporting the findings of this study are available within the paper, its Supplementary Information files, and/or the Source Data file. Images of uncropped SDS PAGE analyses are provided in Supplementary Fig. 6. Source data in figures are provided in Supplementary Data 1. Further information and requests for resources and reagents are available from the corresponding author on reasonable requests or from standard commercial sources as specified in the Methods section.
