# Supplementary material for: ATP-binding and hydrolysis of human NLRP3
Source: Commun Biol. 2022 Nov 3;5:1176. doi: 10.1038/s42003-022-04120-2 (PMC9633759; doi:10.1038/s42003-022-04120-2)
Supplement: Supplementary file 1 — Supplementary Information [file 42003_2022_4120_MOESM1_ESM.pdf]

Supplementary Information

ATP-binding and hydrolysis of human NLRP3

Rebecca Brinkschulte[1], David M. Fußhöller[1], Florian Hoss[2], Juan F. Rodríguez-Alcázar[2], Mario A. Lauterbach[2], Carl-Christian Kolbe[2], Melanie Rauen[1], Semra Ince[3], Christian Herrmann[3], Eicke Latz[2] & Matthias Geyer[1,*]

[1] Institute of Structural Biology, University of Bonn, Venusberg-Campus 1, 53127 Bonn, Germany.
[2] Institute of Innate Immunity, University of Bonn, Venusberg-Campus 1, 53127 Bonn, Germany.
[3] Physical Chemistry I, Ruhr University Bochum, 44780 Bochum, Germany.

* Correspondence should be addressed to: matthias.geyer@uni-bonn.de

Content:

Supplementary Figures 1–6

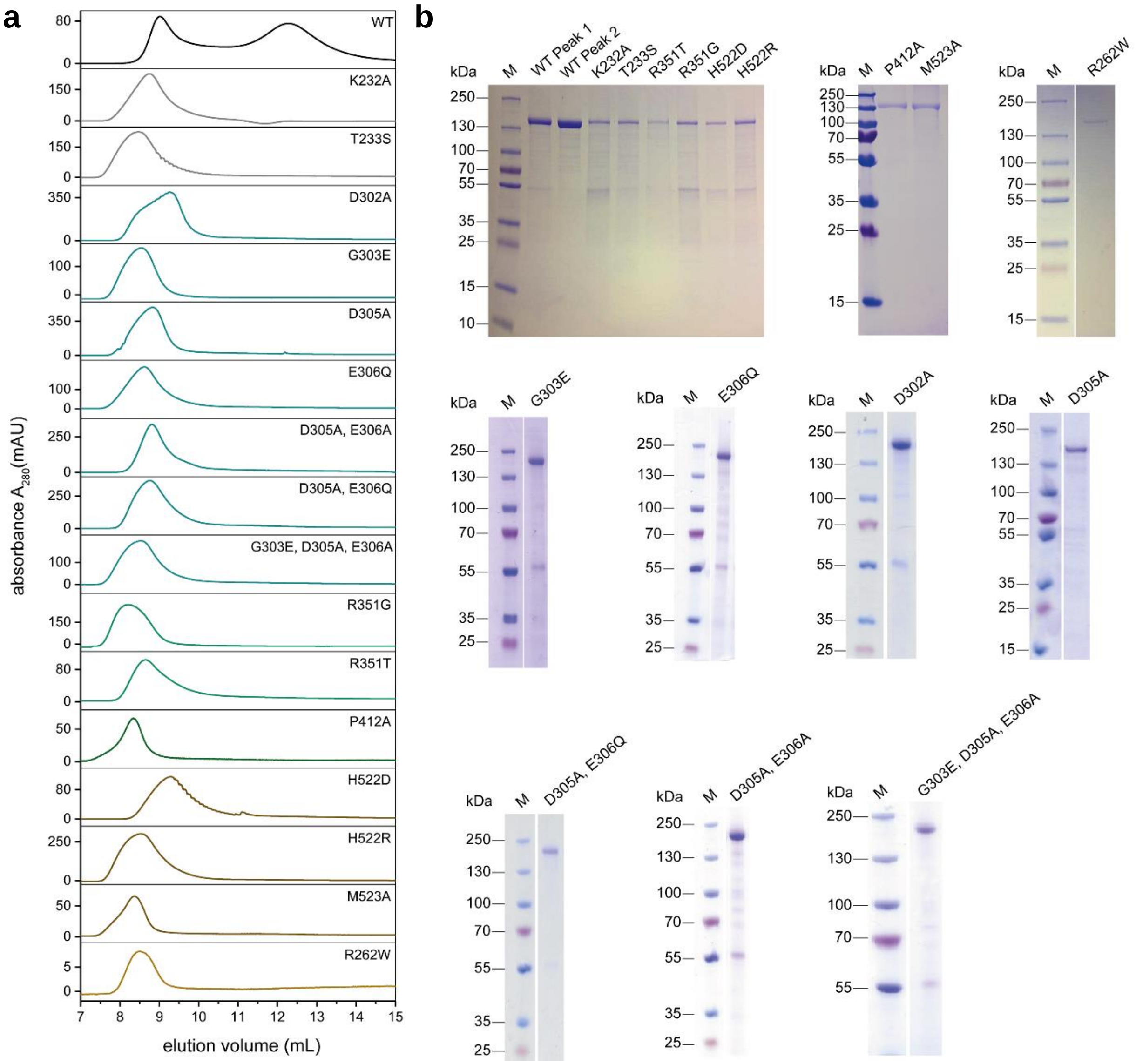

Supplementary Fig. 1 | Elution profiles and homogeneity of MBP-NLRP3 proteins. a, Gel filtration chromatograms of MBP-NLRP3 wild type protein and ATP-binding site mutants. The x-axis shows the elution volume (mL) and the y-axis displays the absorbance at 280 nm in mAU. Point mutations are indicated for each chromatogram. The elution profiles were recorded at room temperature. **b**, Coomassie stained SDS-PAGE analysis showing MBP-NLRP3 ATP-binding site variants after gel filtration.

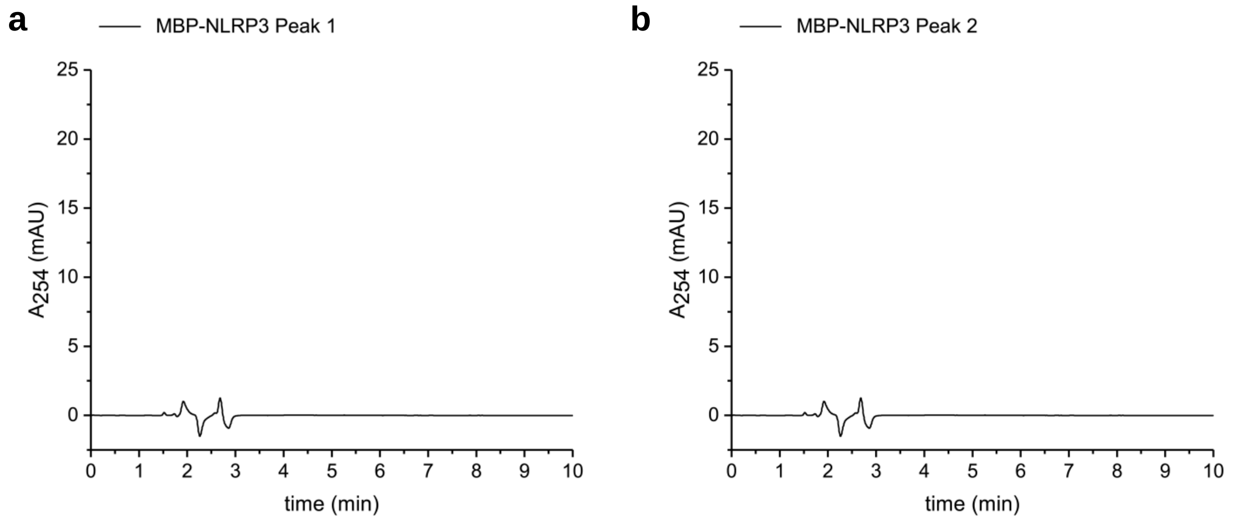

Supplementary Fig. 2 | Recombinant NLRP3 protein purified by affinity chromatography is not loaded with adenosine nucleotides. a, RP-HPLC run of 100 µM MBP-NLRP3 (wt, fl) peak 1 protein at 25°C purified w/o ADP or ATP in the buffer solutions does not exhibit any bound nucleotide. **b**, RP-HPLC run of 100 µM MBP-NLRP3 (wt, fl) peak 2 protein at 25°C purified w/o ADP or ATP in the buffer solutions does not exhibit any bound nucleotide.

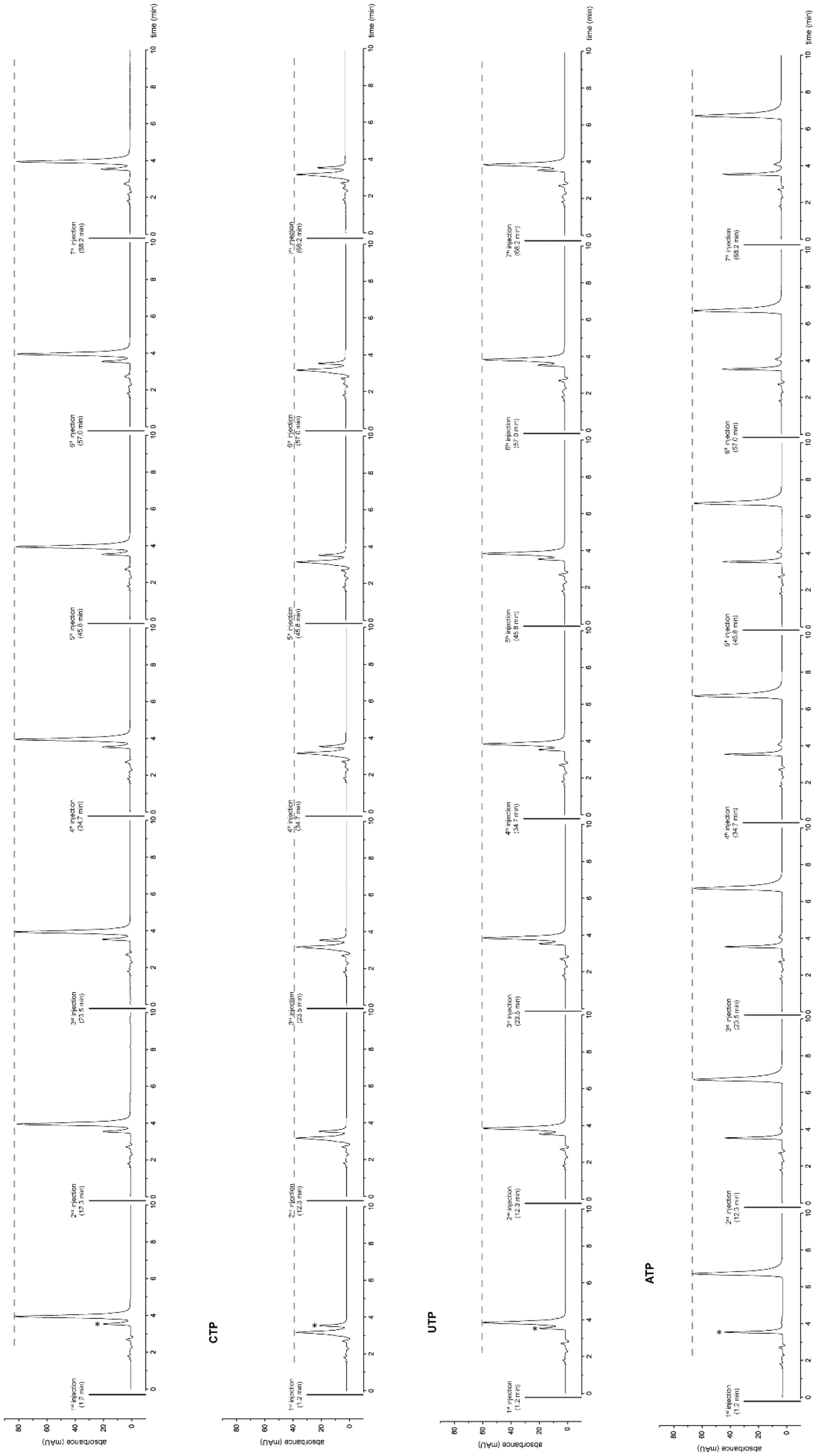

Supplementary Fig. 3 | Time course experiments of nucleotide hydrolysis by NLRP3. RP-HPLC runs of 3 µM MBP-NLRP3 (wt, peak 1) protein with either 100 µM GTP, 100 µM CTP or 100 µM UTP and 5 mM Mg^{2+} at 25°C (top three panels). Every 10 min, a sample was injected to the C18 column. As control, 100 µM ATP without Mg^{2+} was applied (bottom panel).

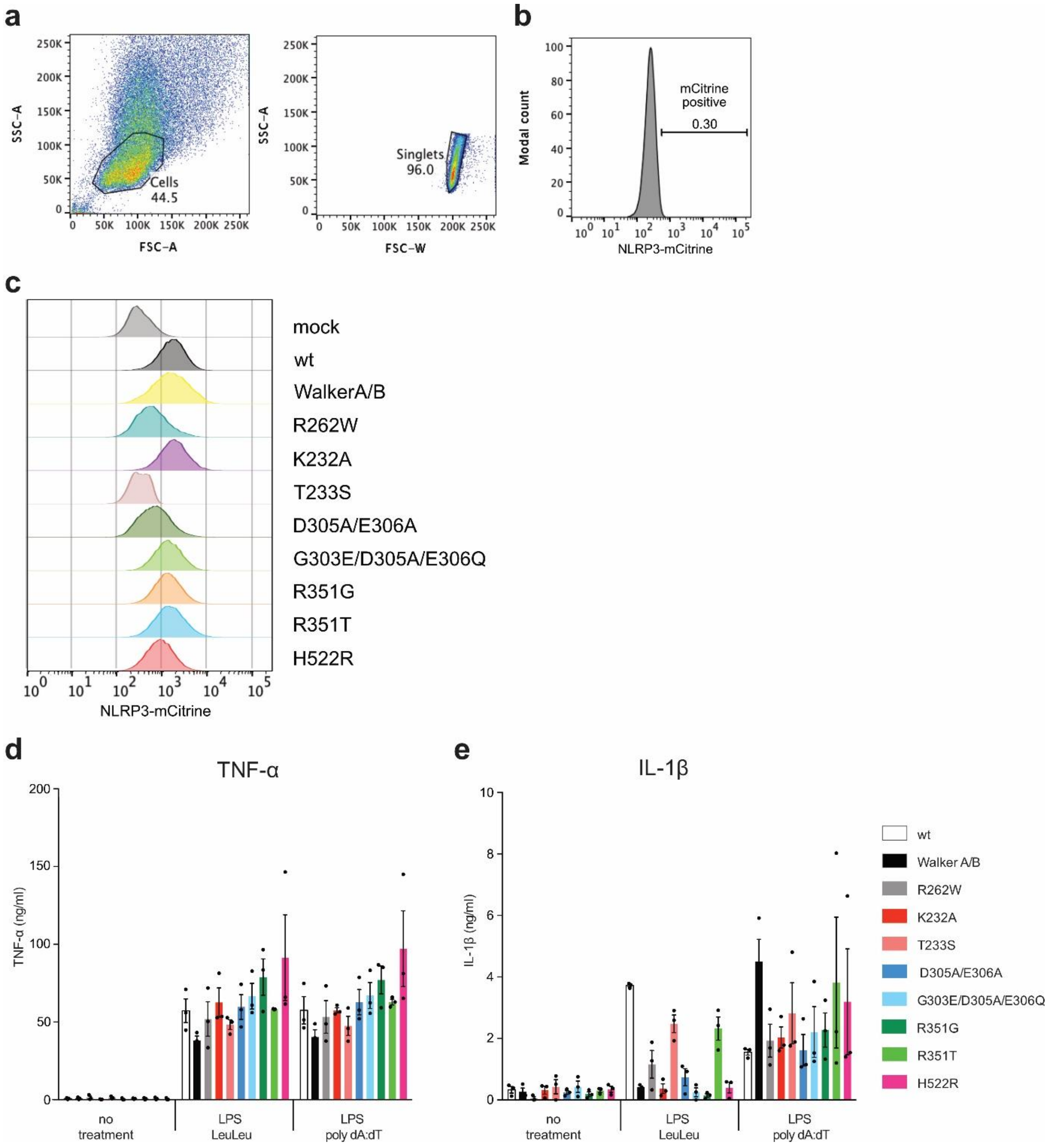

Supplementary Fig. 4 | Reconstitution of NLRP3-deficient macrophages with NLRP3-mCitrine variants. NLRP-deficient immortalized macrophages were retrovirally reconstituted with wildtype NLRP3-mCitrine or indicated variants to generate stable cell lines**. a** and **b**, Gating strategy during cell sorting of the cell lines generated over-expressing different variants of NLRP3. **c**, Expression levels of NLRP3-mCitrine for the cell lines generated. **d**, TNF-α and **e**, IL-1β secretion of unstimulated immortalized macrophages or LPS primed immortalized macrophages stimulated with the NLRP3 activator LeuLeu or the AIM2 activator poly dA:dT. Means +SEM of pooled data from 3 independent experiments are presented.

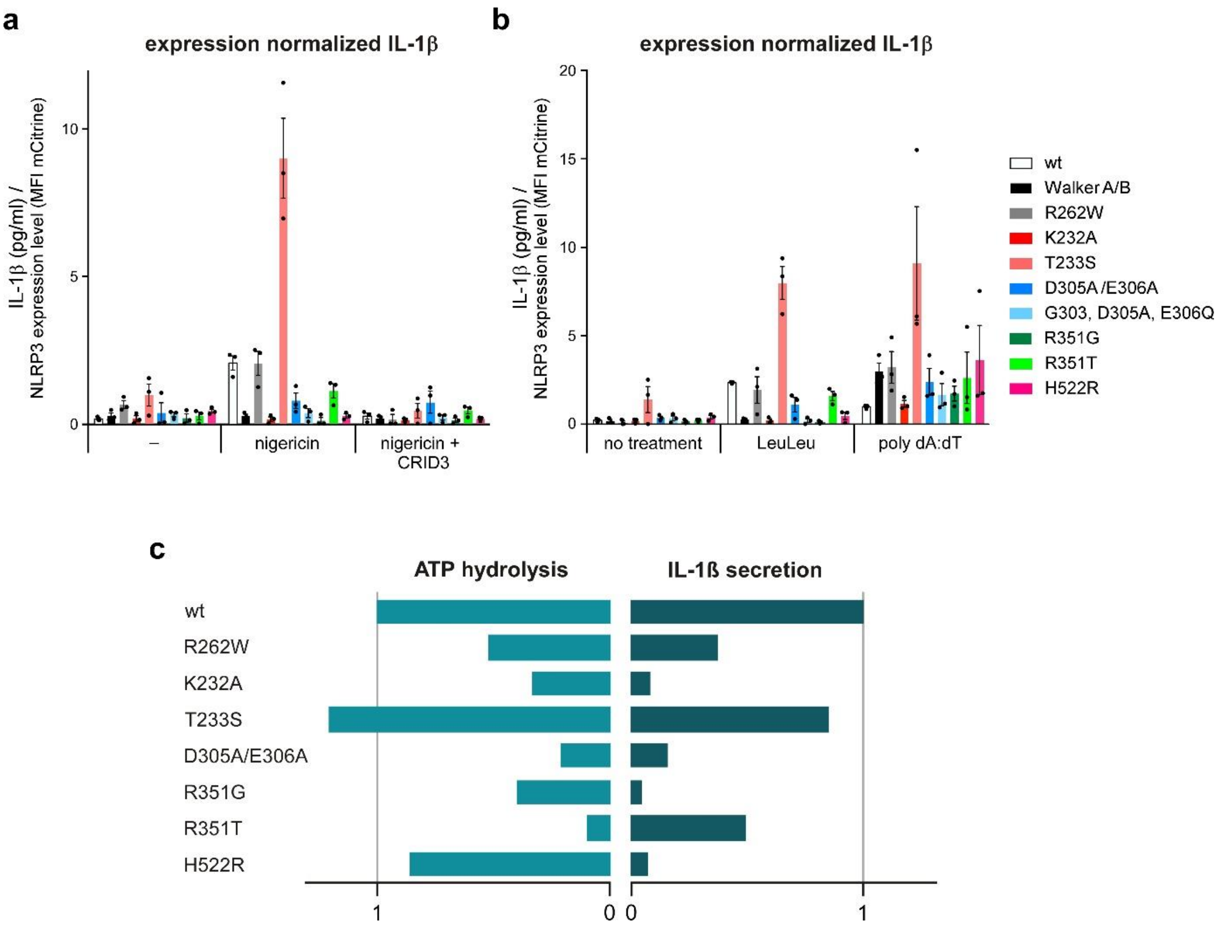

Supplementary Fig. 5 | IL-1β secretion in relation to ATP-hydrolysis. a and **b**, IL-1β secretion (pg/ml) normalized to the expression level of the respective NLRP3 variant (FACS, MFI of mCitrine) at the time of the experiment. Data corresponds to Fig. 5d and Suppl. Fig 4c and e. Means +SEM of pooled data from 3 independent experiments are presented. **c**, IL-1β secretion in relation to ATP-hydrolysis of wild type NLRP3 and variants. The ATP-hydrolysis reaction of full length MBP-NLRP3 and protein variants (always peak 1) was analyzed over time by RP-HPLC using 3 µM NLRP3 protein, 100 µM ATP and 5 mM Mg^{2+} at 25°C. The resulting turnover numbers (min^{-1}) for the indicated MBP-NLRP3 protein variants (Table 1) were normalized to turnover numbers derived for wild type NLRP3 protein. Mean values of IL-1β secretion (ng/ml) of LPS-primed immortalized macrophages stimulated with nigericin was normalized to the respective wt control.

a

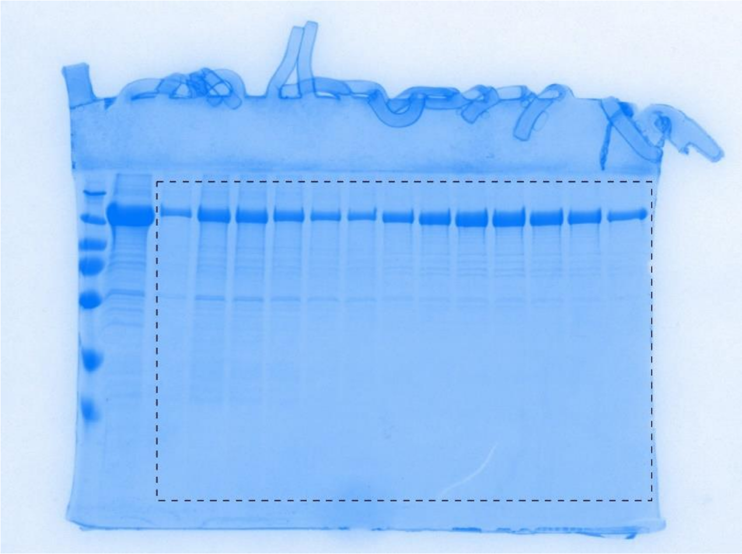

b

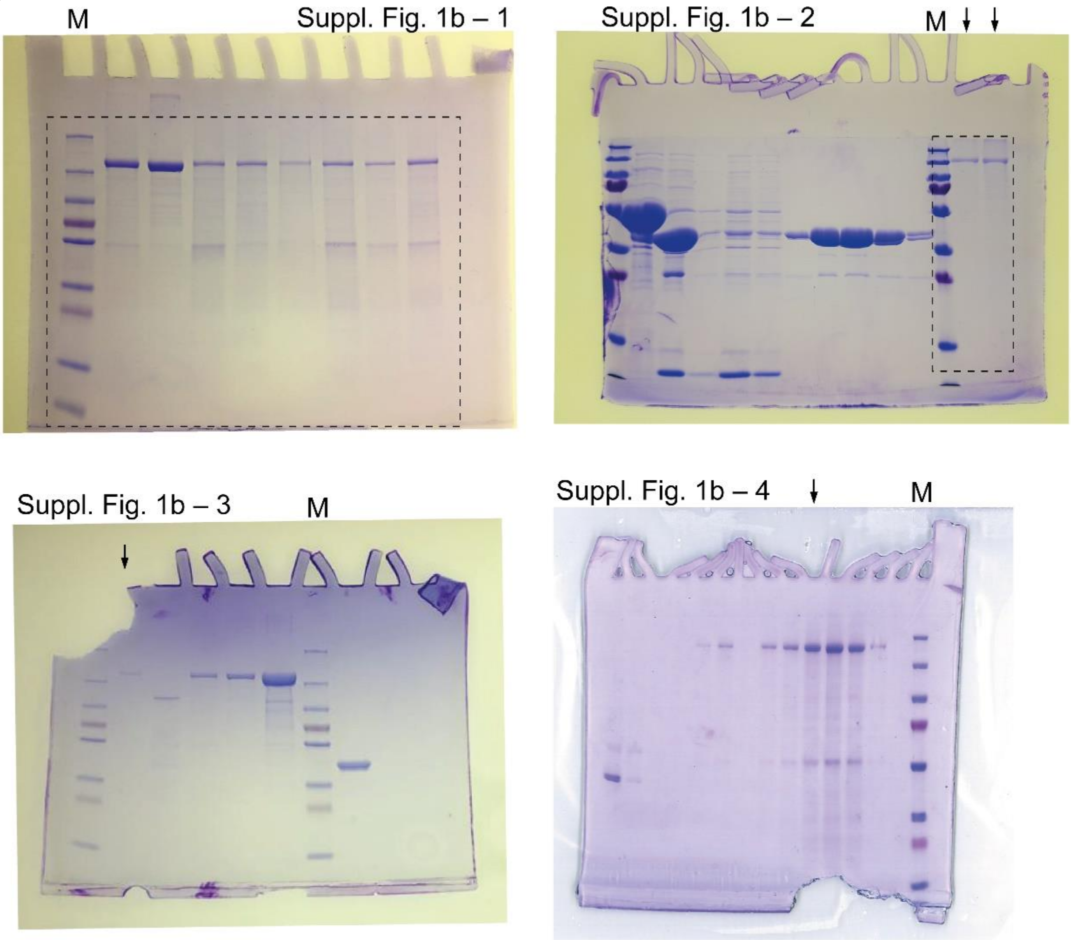

Supplementary Fig. 6 | Original images of Coomassie-stained SDS PAGE analysis experiments. **a**, Original image of the SDS PAGE analysis shown in Fig. 1b. **b**, Original images of SDS PAGE analyses shown in Supplementary Fig. 1b. Uncropped images of the first four Coomassie-stained gels are provided: Supplementary Fig 1b – 1, upper left gel; 2 (P412A, M523A); 3 (R262W); 4 (G303E).

b (cont.)

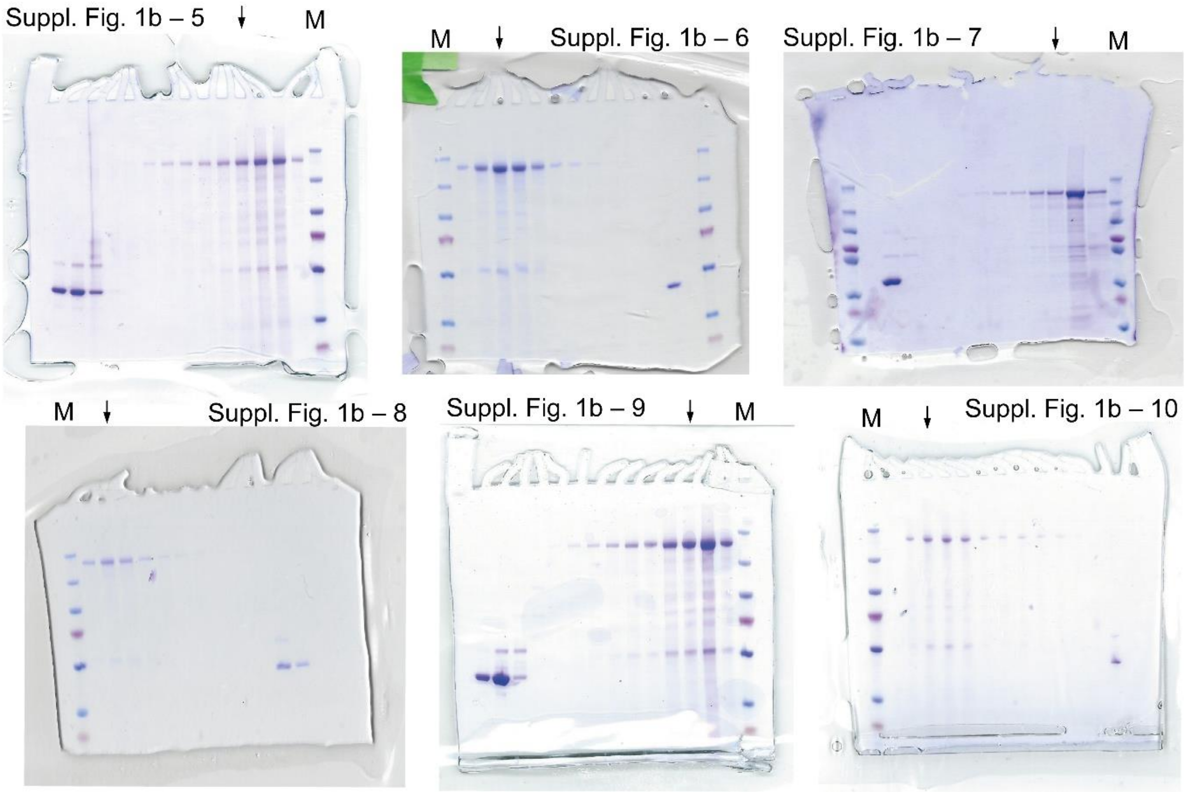

Supplementary Fig. 6 | b (cont.), Original images of SDS PAGE analyses shown in Supplementary Fig. 1b. Uncropped images of the Coomassie-stained gels 5 (E306Q), 6 (D302A), 7 (D305A), 8 (D305A/E306Q), 9 (D305A/E306A), and 10 (G303E/D305A/E306A) are shown.
