# Supplementary material for: ATP-binding and hydrolysis of human NLRP3
Source: Commun Biol. 2022 Nov 3;5:1176. doi: 10.1038/s42003-022-04120-2 (PMC9633759; doi:10.1038/s42003-022-04120-2)
Supplement: Supplementary file 2 — Description of Additional Supplementary Files [file 42003_2022_4120_MOESM2_ESM.pdf]

Description of Additional Supplementary Files

File name: Supplementary Data 1

Description: Source data to Figures 1c, 1d, 2a, 2b, 2c, 2d, 3b, 3c, 4a, 4b, 4c, 4d, 5a, 5b, 5c, 5d, 6a, and 6b in an Excel file.
