# Supplementary material for: ATP-binding and hydrolysis of human NLRP3
Source: Commun Biol. 2022 Nov 3;5:1176. doi: 10.1038/s42003-022-04120-2 (PMC9633759; doi:10.1038/s42003-022-04120-2)
Supplement: Supplementary file 4 — Reporting Summary [file 42003_2022_4120_MOESM4_ESM.pdf]

nature research

Corresponding author(s): Matthias Geyer

Last updated by author(s): Oct 5, 2022

Reporting Summary

Nature Research wishes to improve the reproducibility of the work that we publish. This form provides structure for consistency and transparency in reporting. For further information on Nature Research policies, see our Editorial Policies and the Editorial Policy Checklist.

Statistics

For all statistical analyses, confirm that the following items are present in the figure legend, table legend, main text, or Methods section.

n/a	Confirmed	
☐	☒	The exact sample size (*n*) for each experimental group/condition, given as a discrete number and unit of measurement
☐	☒	A statement on whether measurements were taken from distinct samples or whether the same sample was measured repeatedly
☐	☒	The statistical test(s) used AND whether they are one- or two-sided<br>*Only common tests should be described solely by name; describe more complex techniques in the Methods section.*
☐	☒	A description of all covariates tested
☐	☒	A description of any assumptions or corrections, such as tests of normality and adjustment for multiple comparisons
☐	☒	A full description of the statistical parameters including central tendency (e.g. means) or other basic estimates (e.g. regression coefficient) AND variation (e.g. standard deviation) or associated estimates of uncertainty (e.g. confidence intervals)
☒	☐	For null hypothesis testing, the test statistic (e.g. F, t, r) with confidence intervals, effect sizes, degrees of freedom and P value noted<br>*Give P values as exact values whenever suitable.*
☒	☐	For Bayesian analysis, information on the choice of priors and Markov chain Monte Carlo settings
☒	☐	For hierarchical and complex designs, identification of the appropriate level for tests and full reporting of outcomes
☒	☐	Estimates of effect sizes (e.g. Cohen's *d*, Pearson's *r*), indicating how they were calculated

Our web collection on statistics for biologists contains articles on many of the points above.

Software and code

Policy information about availability of computer code

Data collection	Chromatography data were collected in individual ion-paired reverse phase HPLC measurements, using a Chromolith Performance RP-18 endcapped 100-4.6 HPLC column and the corresponding guard cartridge Chromolith RP-18 (Merck, Darmstadt, Germany). Measurements were performed using an Agilent 1260 infinity PSS bio-inert HPLC System (Agilent Technologies, Inc., Santa Clara, USA).
Data analysis	For the analysis of HPLC data, the peak borders were set manually and the peak areas were integrated using the internal PSS software. Regression curves represent exponential growth fittings.

For manuscripts utilizing custom algorithms or software that are central to the research but not yet described in published literature, software must be made available to editors and reviewers. We strongly encourage code deposition in a community repository (e.g. GitHub). See the Nature Research guidelines for submitting code & software for further information.

Data

Policy information about availability of data

All manuscripts must include a data availability statement. This statement should provide the following information, where applicable:

- Accession codes, unique identifiers, or web links for publicly available datasets
- A list of figures that have associated raw data
- A description of any restrictions on data availability

The authors declare that all data supporting the findings of this study are available within the paper, its Supplementary Information files and/or the Source Data file. All unique materials used are readily available from the authors upon reasonable request or from standard commercial sources as specified in the Methods section.

April 2020

Field-specific reporting

Please select the one below that is the best fit for your research. If you are not sure, read the appropriate sections before making your selection.

☒ Life sciences ☐ Behavioural & social sciences ☐ Ecological, evolutionary & environmental sciences

For a reference copy of the document with all sections, see nature.com/documents/nr-reporting-summary-flat.pdf

Life sciences study design

All studies must disclose on these points even when the disclosure is negative.

Sample size	Protein purifed was set to a fixed concentration, as described in the materials and methods section. Cell density in cellular experiments is indicated.
Data exclusions	No data were excluded from the analyses.
Replication	For all measurements of hydrolysis activity, individual protein preparations were analyzed (n=1). Cell experimental data was derived from sample sizes of n=5, as detailed in the Methods or Figure Legends.
Randomization	Randomiziation is not applicable for this study.
Blinding	Blinding is not applicable for this study.

Reporting for specific materials, systems and methods

We require information from authors about some types of materials, experimental systems and methods used in many studies. Here, indicate whether each material, system or method listed is relevant to your study. If you are not sure if a list item applies to your research, read the appropriate section before selecting a response.

Materials & experimental systems

n/a	Involved in the study	
☒	☐	Antibodies
☐	☒	Eukaryotic cell lines
☒	☐	Palaeontology and archaeology
☒	☐	Animals and other organisms
☒	☐	Human research participants
☒	☐	Clinical data
☒	☐	Dual use research of concern

Methods

n/a	Involved in the study	
☒	☐	ChIP-seq
☐	☒	Flow cytometry
☒	☐	MRI-based neuroimaging

Eukaryotic cell lines

Policy information about cell lines

Cell line source(s)	Sf9 cells: ThermoFisher, Catalogue Number 12659017. Murine immortalized macrophages NLRP3 -/-: Latz Lab, UMASS Worcester, USA, produced as described in Hornung, V. et al. 2008 (PMID: 18604214). HeLa cells stably expressing ASC-mTurquoise were produced as described in Cardona G, et al. 2018 (PMID: 29177858).
Authentication	Cell lines were verfieid by manufacturer's website and cellular identity was regularly checked by morphology.<br>THP-1 cells: (ATCC) https://www.atcc.org/products/tib-202<br>HEK293T cells: (ATCC) https://www.atcc.org/products/crl-3216<br>Sf9 cells: (ThermoFisher) https://www.thermofisher.com/order/catalog/product/12659017#/12659017<br>FreeStyle 293-F cells: https://www.thermofisher.com/order/catalog/product/R79007<br>HeLa cells: (ATCC) https://www.lgcstandards-atcc.org/products/all/CCL-2.aspx?geo_country=de
Mycoplasma contamination	Cell lines were tested mycoplasma negative through PCR.
Commonly misidentified lines (See ICLAC register)	No commonly misidentified cell lines were used.

Flow Cytometry

Plots

Confirm that:

- [x] The axis labels state the marker and fluorochrome used (e.g. CD4-FITC).
- [x] The axis scales are clearly visible. Include numbers along axes only for bottom left plot of group (a 'group' is an analysis of identical markers).
- [ ] All plots are contour plots with outliers or pseudocolor plots.
- [x] A numerical value for number of cells or percentage (with statistics) is provided.

Methodology

Sample preparation	Cells were harvested and analyzed for the expression of the respective NLRP3-fluorescence proteins.
Instrument	BD Aria, MACS-Quant VYB
Software	FlowJo, FACS-Diva (BD)
Cell population abundance	Sorted cell lines were afterwards controlled for expression as indicated.
Gating strategy	All cells were included in the analysis and doublets were excluded via FSC-W. Sorting gates were based on cells not expressing the fluorescent protein.

- [x] Tick this box to confirm that a figure exemplifying the gating strategy is provided in the Supplementary Information.
